# Supplementary material for: Post-Harvest Treatment with Methyl Jasmonate Impacts Lipid Metabolism in Tomato Pericarp (Solanum lycopersicum L. cv. Grape) at Different Ripening Stages
Source: Foods. 2021 Apr 16;10(4):877. doi: 10.3390/foods10040877 (PMC8073352; doi:10.3390/foods10040877)
Supplement: Supplementary file 1 [file foods-10-00877-s001.zip › foods-1173705-SI.pdf]

Table S1. Carotenoids contents (μg.g^{-1} FW) in tomato pericarp (*Solanum lycopersicum* L. cv. Grape) exposed to 1-methylcyclopropene (MCP) and both 1-methylcyclopropene and methyl jasmonate (MCP+ MeJA) treatments at 04, 10 and 21 days after harvest (DAH) detected by high performance liquid chromatography (HPLC).

Metabolite	**04 DAH**			**10 DAH**			**21 DAH**		
	**CTRL**	**MCP**	**MCP + MeJA**	**CTRL**	**MCP**	**MeJA + MCP**	**CTRL**	**MCP**	**MCP + MeJA**
Lycopene	15.4 ± 0.43^{g}	0.52 ± 0.01^{h}	0.63 ± 0.01^{h}	487.1 ± 4.10^{c}	61.1 ± 0.59^{f}	81.9 ± 0.19^{e}	653.6 ± 0.82^{b}	234.8 ± 0.66^{d}	712.5 ± 0.35^{a}
β-carotene	3.87 ± 0.02^{d}	2.57 ± 0.02^{g}	2.72 ± 0.01^{f}	5.32 ± 0.10^{c}	2.68 ± 0.07^{fg}	2.97 ± 0.02^{e}	7.23 ± 0.01^{b}	3.02 ± 0.01^{c}	8.02 ± 0.01^{a}
Lutein	2.43 ± 0.02^{g}	2.07 ± 0.01^{i}	2.31 ± 0.01^{h}	3.27 ± 0.06^{c}	2.56 ± 0.04^{f}	2.88 ± 0.03^{d}	3.93 ± 0.05^{b}	2.75 ± 0.04^{e}	4.67 ± 0.03^{a}
Total	21.7 ± 0.42^{g}	5.17 ± 0.01^{h}	5.66 ± 0.03^{h}	495.6 ± 4.10^{c}	66.3 ± 0.63^{f}	87.8 ± 0.22^{e}	664.7 ± 0.85^{b}	240.8 ± 0.68^{d}	725.1 ± 0.35^{a}

CTRL: Control fruits. Different superscript letters (a-h) indicate statistical significance ($P < 0.05$) at the same line (mean ± standard deviation. $n = 4$).

Table S2. Tocopherols, phytol and phytosterols in tomato pericarp (*Solanum lycopersicum* L. cv. Grape) exposed to 1-methylcyclopropene (MCP) and both 1-methylcyclopropene and methyl jasmonate (MCP+ MeJA) treatments at 04, 10 and 21 days after harvest (DAH) detected by gas chromatography-mass spectrometry (GC-MS).

Metabolite	**04 DAH**			**10 DAH**			**21 DAH**		
	**CTRL**	**MCP**	**MCP + MeJA**	**CTRL**	**MCP**	**MCP + MeJA**	**CTRL**	**MCP**	**MCP + MeJA**
A) Tocopherols									
α-tocopherol	152,7 ± 14,5^{d}	32,1 ± 1,41^{e}	37,1 ± 6,01^{e}	531,9 ± 12,1^{a}	42,7 ± 4,84^{e}	193,4 ± 9,80^{c}	375,8 ± 1,57^{b}	137,2 ± 1,41^{d}	535,8 ± 40,2^{a}
β-tocopherol	14,1 ± 0,27^{d}	0,98 ± 0,13^{g}	1,15 ± 0,18^{g}	62,3 ± 2,15^{a}	2,66 ± 0,12^{g}	6,85 ± 0,10^{e}	31,6 ± 0,23^{c}	4,70 ± 0,02^{f}	44,8 ± 0,03^{b}
γ-tocopherol	11,6 ± 0,49^{d}	1,91 ± 0,15^{g}	2,03 ± 0,05^{g}	14,6 ± 0,38^{c}	2,71 ± 0,09^{g}	8,71 ± 0,16^{e}	18,9 ± 0,72^{b}	3,71 ± 0,12^{f}	22,7 ± 0,79^{a}
Total	178,4 ± 15,1^{cd}	34,9 ± 0,86^{e}	40,3 ± 0,99^{e}	608,9 ± 14,6^{a}	48,11 ± 4,77^{e}	208,9 ± 6,07^{c}	426,4 ± 1,45^{b}	145,6 ± 1,67^{e}	603,2 ± 39,8^{a}
B) Acyclic diterpenoids									
Phytol	21,3 ± 2,25^{a}	10,9 ± 0,21^{b}	19,5 ± 1,05^{a}	9,54 ± 0,18^{bc}	9,80 ± 0,59^{b}	19,9 ± 0,18^{a}	6,75 ± 0,04^{d}	7,19 ± 0,14^{d}	7,52 ± 0,16^{cd}
C) Phytosterols									
β-sitosterol	113,2 ± 7,14^{f}	21,9 ± 0,61^{h}	29,7 ± 0,89^{h}	410,2 ± 10,9^{b}	79,5 ± 4,59^{g}	151,9 ± 2,45^{e}	282,7 ± 1,32^{d}	171,1 ± 16,5^{c}	487,6 ± 15,3^{a}
Stigmasterol	227,3 ± 10,5^{f}	50,9 ± 1,04^{d}	60,5 ± 1,38^{e}	344,1 ± 8,75^{e}	49,6 ± 1,76^{b}	68,9 ± 0,19^{a}	400,8 ± 1,71^{d}	164,5 ± 4,68^{c}	535,6 ± 10,6^{ab}
Stigmastadienol	18,2 ± 0,26^{d}	2,10 ± 0,13^{h}	6,47 ± 0,21^{f}	27,8 ± 0,68^{c}	5,62 ± 0,11^{g}	7,31 ± 10,18^{e}	34,2 ± 0,16^{b}	7,03 ± 0,13^{ef}	44,9 ± 0,21^{a}
Total	358,7 ± 14,3^{e}	74,9 ± 1,63^{h}	96,7 ± 2,14^{h}	782,0 ± 20,2^{b}	134,8 ± 6,05^{g}	228,2 ± 2,39^{f}	717,6 ± 2,80^{c}	542,7 ± 19,9^{d}	1068,0 ± 25,3^{a}

Values were presented as normalized area by n-tridecane (internal non-polar standard). CTRL: Control fruits. Different superscript letters (a-h) indicate statistical significance ($P < 0.05$) at the same line (mean ± standard deviation, $n = 4$).
